# Fulminant Tetraplegia in a Patient on Infliximab: A Case of Masked Cervical Spinal Epidural Abscess Presenting With Dysphagia

**DOI:** 10.7759/cureus.107859

**Published:** 2026-04-28

**Authors:** Colin Dubois, Pascale Lievens

**Affiliations:** 1 Emergency Department, Centre Hospitalier Régional Sambre et Meuse - Site Meuse (Namur), Namur, BEL

**Keywords:** anti-tnf therapy, cervical spinal epidural abscess, cervical spondylodiscitis, dysphagia, infliximab, mssa, spinal epidural abscess, staphylococcus aureus, tetraplegia

## Abstract

While spinal epidural abscess (SEA) is a well-recognized neurosurgical emergency, its diagnosis remains a formidable challenge when presenting with atypical symptoms such as dysphagia, particularly in patients under anti-tumor necrosis factor (TNF) therapy. This report describes a critical diagnostic trap: a fulminant cervical SEA in a 59-year-old male patient treated with infliximab for ankylosing spondylitis. Despite being afebrile and neurologically intact upon admission, laboratory investigations revealed a massive inflammatory syndrome (C-reactive protein: 282.9 mg/L). His condition rapidly progressed to sudden tetraplegia during diagnostic imaging. MRI confirmed C3-C4 spondylodiscitis with a massive prevertebral and spinal epidural abscess causing critical cord compression (5 mm residual canal). The patient underwent emergency C3-C4 anterior discectomy and abscess drainage. Despite the successful eradication of the methicillin-susceptible *Staphylococcus aureus* (MSSA) infection, the patient suffered permanent neurological deficits. This case highlights the potent masking effect of anti-TNF therapy on systemic inflammatory signs and the diagnostic challenge of SEA presenting as dysphagia. It underscores that in immunocompromised patients, the absence of fever must not rule out severe infection when neck pain and elevated inflammatory markers are present. Clinicians must maintain a low threshold for emergency MRI, as the therapeutic window for intervention is exceptionally narrow once fulminant neurological deficits manifest.

## Introduction

Spinal epidural abscess (SEA) is a well-recognized and clinically critical neurosurgical emergency. Despite its established pathophysiology and advancements in diagnostic imaging, it remains a formidable diagnostic challenge due to its frequently non-specific initial presentation. The classic clinical triad of fever, spinal pain, and neurological deficit is observed in fewer than 15% of patients at the time of first evaluation [1].

The risk of developing SEA is significantly higher in patients with predisposing factors such as diabetes mellitus or chronic immunosuppression [2]. In recent years, the widespread use of biological therapies, particularly tumor necrosis factor-alpha (TNF-α) inhibitors like infliximab, has introduced a critical diagnostic pitfall. These agents can profoundly attenuate the host’s systemic inflammatory response, often masking cardinal signs of infection such as fever and leukocytosis [3]. Consequently, severe pyogenic infections can progress silently until catastrophic neurological compromise occurs.

The novelty of the present case lies in the intersection of a misleading clinical presentation, dysphagia, and the potent masking effect of anti-TNF therapy. While cervical SEA is less common than lumbar localization, it is particularly dangerous due to the narrowness of the spinal canal and the risk of rapid tetraplegia. In the cervical region, an abscess can present with atypical symptoms such as dysphagia, which often leads to initial misdiagnosis as a localized oropharyngeal infection [1]. Because the prognosis is strictly dependent on the neurological status at the time of surgery, recognizing this specific clinical scenario as a "diagnostic trap" is paramount to avoid permanent disability [4]. This report aims to illustrate the rapid progression from apparent clinical stability to fulminant tetraplegia and emphasize the exceptionally narrow therapeutic window in such patients.

## Case presentation

Initial assessment

A 59-year-old male patient presented to the emergency department reporting bilateral neck pain radiating to the shoulders for three days and progressive dysphagia to solids. His medical history was significant for ankylosing spondylitis treated with infliximab every 12 weeks. On admission, the patient was afebrile (36.4°C) and hemodynamically stable. The initial neurological examination was unremarkable, with a Glasgow Coma Scale of 15/15 [5] and no motor or sensory deficits (5/5 power in all limbs) [6]. However, the otorhinolaryngology examination (ENT) noted retropharyngeal asymmetry and purulent secretions.

Diagnostic investigations

Laboratory results revealed a massive inflammatory syndrome with a white blood cell count (WBC) of 13,790/µl and a C-reactive protein (CRP) of 282.9 mg/L. Complete laboratory results on admission are summarized in Table 1.

**Table 1:** Laboratory results on admission

Parameter	Result	Reference range	Units
Hematology			
White blood cells (WBC)	13.79	4.0 – 11.0	10³/µL
Neutrophils (%)	89.3	49.2 – 78.4	%
Neutrophils (Absolute)	12.31	1.4 – 7.7	10³/µL
Lymphocytes (Absolute)	0.51	1.0 – 4.8	10³/µL
Hemoglobin	14.9	13.0 – 18.0	g/dL
Platelets	162	150 – 400	10³/µL
Chemistry & Inflammation			
C-reactive protein (CRP)	282.9	0.0 – 5.0	mg/L
Creatinine	0.68	0.70 – 1.20	mg/dL
Alanine aminotransferase (ALT/GPT)	58	0 – 41	U/L
Sodium	144	135 – 145	mmol/L
Potassium	3.87	3.5 – 5.0	mmol/L

Initial blood cultures were drawn, which subsequently confirmed methicillin-susceptible *Staphylococcus aureus* (MSSA) bacteremia.

A systematic search for the primary portal of entry was performed. The patient had no history of recent trauma, skin lesions, dental work, or intravenous drug use. No clinical or echocardiographic evidence of endocarditis was identified. Therefore, the infection was considered to have originated from an occult hematogenous seeding of the C3-C4 disc space, a process potentially favored by the systemic 'masking' and immunosuppressive effects of long-term infliximab therapy.

**Figure 1:**
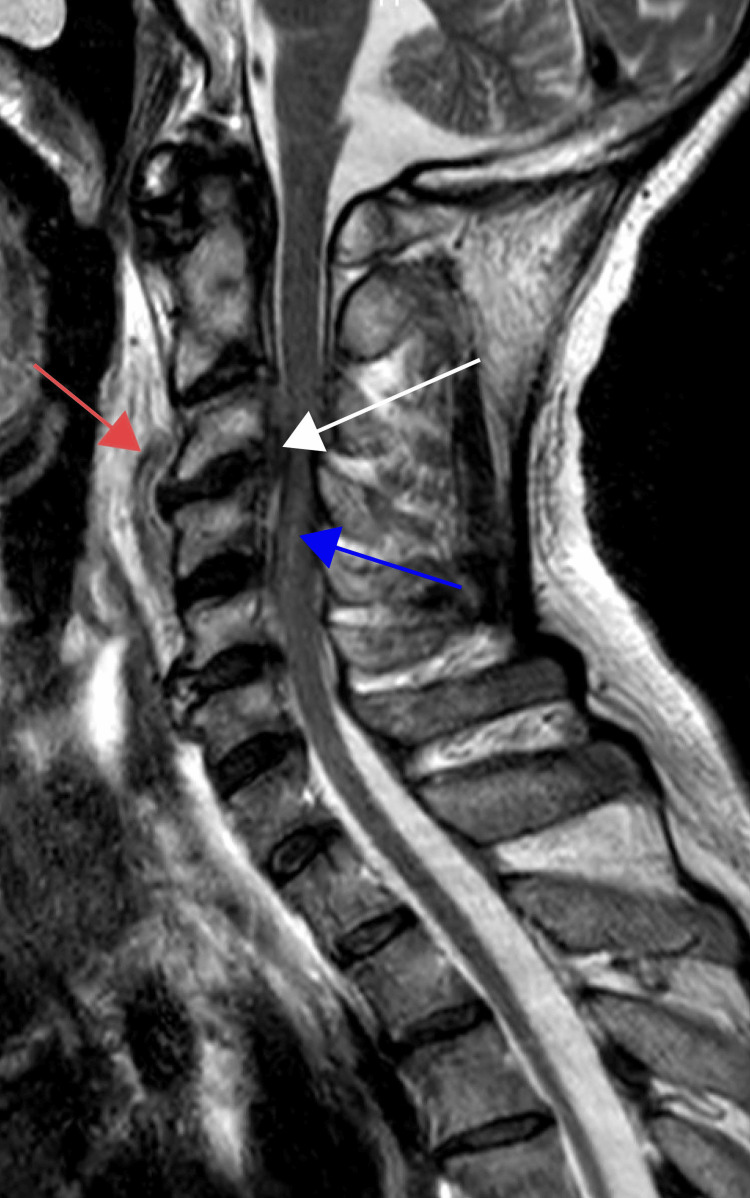
Midsagittal T2-weighted MRI of the cervical spine The image demonstrates extensive C3-C4 spondylodiscitis with significant inflammatory changes. A large prevertebral abscess (red arrow) is visible, extending from C2 to C6 and causing anterior displacement of the retropharyngeal soft tissues. At the C3-C4 level, a critical anterior spinal epidural abscess (white arrow) is seen, leading to severe spinal cord compression with a residual spinal canal diameter of only 5 mm. A diffuse intramedullary T2 hypersignal is present from C3 to C6 (blue arrow), consistent with acute compressive myelopathy.

A cervical CT scan demonstrated a 12 mm thick prevertebral collection extending from C2 to C6. Given the risk of airway compromise and neurological involvement, an urgent MRI was performed. This confirmed the presence of a massive 60 mm cranio-caudal prevertebral collection and a 55 mm anterior SEA centered at the C3-C4 level. The epidural component resulted in critical narrowing of the spinal canal, with a residual diameter of only 5 mm, causing severe mechanical compression of the spinal cord. Furthermore, extensive intramedullary T2-weighted hypersignal was noted from C3 to C6, consistent with compressive myelopathy and significant cord edema. These findings were associated with C3-C4 spondylodiscitis, characterized by disc space destruction and evidence of posterior longitudinal ligament involvement, which was later confirmed intraoperatively (Figure 1).

Clinical course and management

During the MRI procedure, the patient's condition rapidly deteriorated to sudden tetraplegia. He was immediately transferred for emergency surgery. Surgeons performed an emergency C3-C4 anterior cervical discectomy and drainage of a premedullary extradural abscess. Intraoperative findings revealed significant inflammatory swelling of the prevertebral tissues and frank pus within the disc space, confirming the destruction of the posterior longitudinal ligament. The abscess extended into the premedullary and retrocorporeal spaces at the C3-C4 levels. After thorough debridement and isobetadine irrigation, a flexible drain was placed. Notably, no osteosynthesis or internal fixation was performed during this procedure due to the active infection and the risk of biofilm formation on foreign hardware; consequently, a rigid cervical collar was applied for external spinal stabilization. Specimens were collected for microbiological analysis.

Following the identification of MSSA in both blood and surgical cultures, antibiotic therapy was switched to intravenous flucloxacillin. Due to persistent bacteremia on hospital day three, clindamycin was added for a seven-day course.

The sequence of clinical events, illustrating the rapid progression from stable presentation to surgical intervention, is summarized in Table 2.

**Table 2:** Timeline of clinical events ED: Emergency department; GCS: Glasgow coma scale.

Time point	Event	Key clinical findings & actions
3 days before admission	Symptom onset	Bilateral neck pain and progressive dysphagia.
Day 1: Admission (T=0)	ED Presentation	Patient afebrile and neurologically intact (GCS 15, 5/5 power).
Day 1: +1-2 hours	Initial Investigations	Discovery of massive inflammatory syndrome (CRP: 282.9 mg/L); CT scan performed.
Day 1: +3-4 hours	Urgent MRI	Identification of a 55 mm SEA with critical cord compression (5 mm residual canal).
During MRI procedure	Neurological Deterioration	Sudden progression to fulminant tetraplegia.
Day 1: +5 hours	Emergency Surgery	C3-C4 anterior discectomy and abscess drainage.
Day 1 to Day 3	Targeted Antibiotics	Switch to IV flucloxacillin; addition of clindamycin on day 3.
4 months later	Long-term Follow-up	MRI shows abscess resolution; persistent spastic tetraplegia.

Follow-up and outcome

**Figure 2:**
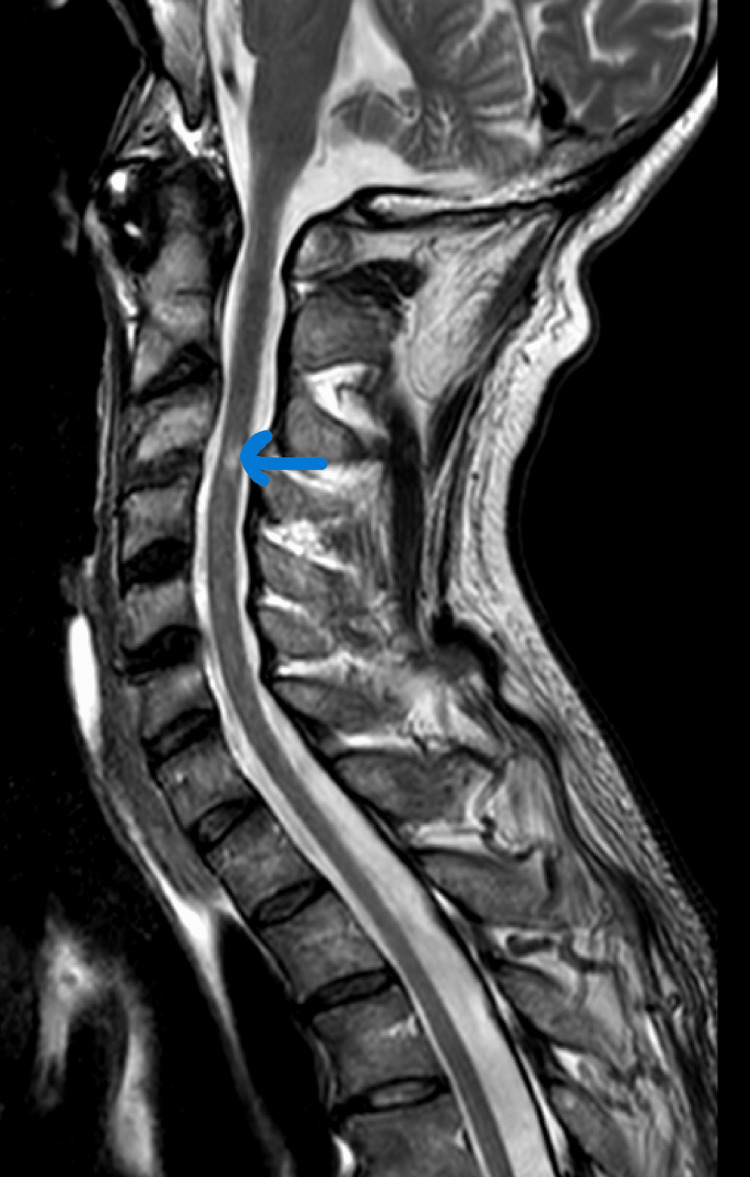
Follow-up sagittal T2-weighted MRI at four months The image demonstrates the complete resolution of the pre-vertebral and epidural abscesses. The C3/C4 disc signal has normalized. A persistent, regressive intramedullary T2 hypersignal (blue arrow) is visible, consistent with chronic compressive myelopathy.

Following surgery, the patient was managed in the intensive care unit (ICU) for 29 days, before being transferred to the neurosurgery ward and subsequently to an intensive rehabilitation center. A follow-up MRI performed four months after admission showed complete resolution of the abscesses (Figure 2). 

To date, the patient remains dependent with spastic tetraplegia and neurogenic bladder dysfunction.

## Discussion

SEA remains a diagnostic challenge, with its incidence rising due to an aging population and the increasing use of immunomodulatory therapies [2]. The classic clinical triad of fever, back pain, and neurological deficit is frequently absent, often leading to misdiagnosis [1].

Limited systemic presentation and the role of anti-TNF therapy

The most striking feature of this case was the absence of fever despite a massive infection. Infliximab significantly alters the systemic immune response by blocking key pro-inflammatory cytokines. These agents can suppress the febrile response and attenuate classic symptoms [3]. In the present case, the role of anti-TNF therapy is best understood not as a complete masking of the infection, but rather as an attenuation of the systemic inflammatory response, specifically the absence of fever. While the patient remained afebrile, the infection was far from silent: the markedly elevated CRP (282.9 mg/L), neutrophilic leukocytosis, and purulent secretions on ENT examination provided clear evidence of a severe pyogenic process. This dissociation represents a critical diagnostic pitfall: the absence of fever alone should not lead to an underestimation of the severity of a spinal infection when other clinical indicators are present.

Dysphagia as a rare sentinel sign

In this case, dysphagia resulted from the mechanical compression of the esophagus by the prevertebral collection. Initial findings of retropharyngeal asymmetry could have been misidentified as a simple deep neck space infection, emphasizing the need to consider spinal pathology in the differential diagnosis of dysphagia in immunocompromised individuals [1]. The differential diagnosis for an adult presenting with acute dysphagia and a prevertebral collection is broad. Primarily, it includes deep neck space infections such as retropharyngeal or parapharyngeal abscesses, which are often secondary to dental infections or upper respiratory tract trauma [1]. Oropharyngeal malignancies or foreign body impaction must also be considered, though they rarely present with such a massive systemic inflammatory response.

Distinguishing between a primary retropharyngeal infection and a cervical SEA presenting with dysphagia is challenging. Primary ENT infections, such as retropharyngeal or parapharyngeal abscesses, typically present with 'local' signs including odynophagia, trismus, or drooling, and are often secondary to dental work or upper respiratory tract trauma. In contrast, when dysphagia originates from spinal pathology, it is often accompanied by 'axial' symptoms, notably severe neck pain and significant restriction of cervical range of motion [1]. In our patient, while the ENT examination showed retropharyngeal asymmetry suggesting a localized infection, the disproportionate intensity of the neck pain and the massive elevation of CRP (282.9 mg/L) acted as critical 'red flags.' These findings suggested that the prevertebral collection was not an isolated ENT process but rather the anterior extension of a deeper, more aggressive spinal infection. This underscores the importance of not stopping at an ENT diagnosis when axial spinal pain is prominent, especially in immunosuppressed patients where the clinical picture is blurred.

To further contextualize our findings, we conducted a literature review of cervical SEA cases presenting with dysphagia, as summarized in Table 3 [7-11].

**Table 3:** Comparison of clinical characteristics and outcomes in reported cases of cervical spinal epidural abscess (SEA) presenting with dysphagia MSSA: methicillin-susceptible *Staphylococcus aureus*.

Study author	Age/Sex	Cervical level(s)	Dysphagia (description)	Proposed mechanism	Organism	Treatment	Outcome
Dubois et al. (Present case)	59/M	C3–C4 (prevert. C2–C6; epidural C3–C4)	Progressive dysphagia to solids; retropharyngeal asymmetry	Contiguous spread; masking by anti-TNF (infliximab)	MSSA	Emergency C3–C4 discectomy, drainage, IV Flucloxacillin	Permanent spastic tetraplegia; radiologic resolution
Ghaly et al. [7]	Adult	C2–C5/6	Progressive dysphagia as main complaint	Anterior collection contiguous with epidural space	None isolated	Prolonged IV antibiotics (conservative)	Clinical recovery
Aydin et al. [8]	N/S	Cervical	Dysphagia as presenting symptom	Anterior/retropharyngeal/epidural involvement	N/S	Individualized management	Illustrative case
Ying et al. [9]	43/M	Paraesophageal/prevert. + intracanalar	Slight/intermittent dysphagia	Direct seeding from esophageal diverticulum	Oral flora	Conservative IV antibiotics	Marked resolution; good recovery
Azam et al. [10]	48/M	C3–C5 (anterior + epidural)	New dysphagia 10 days prior	Spread from retropharyngeal blastomycosis abscess	Blastomyces spp.	Surgical drainage + decompression + Antifungals	Near resolution of symptoms
Cunha et al. [11]	59/M	Prevert. + epidural C3–C7	Dysphagia with neck and neuro signs	Retropharyngeal abscess contiguous to spine	MSSA	Surgical drainage + IV antibiotics	Good clinical/radiologic recovery

This comparison reveals that while dysphagia typically stems from the anterior expansion of the abscess or mechanical compression of the esophagus [7,8], the specific etiology and clinical progression can vary significantly. Mechanisms described in the literature range from direct seeding via esophageal diverticula [9] to rare fungal retropharyngeal involvements [10]. Notably, in several reported cases where patients were not profoundly immunocompromised, earlier clinical suspicion often led to more favorable outcomes [11]. In our patient, however, the synergistic effect of infliximab-induced masking and the high cervical localization (C3-C4) resulted in a uniquely fulminant deterioration, leaving an exceptionally narrow therapeutic window.

This clinical scenario provides a valuable educational insight: it suggests that in the context of biological therapy, traditional 'red flags' for SEA might be insufficiently sensitive. Clinicians must maintain a high index of suspicion for atypical symptoms like dysphagia and rely more heavily on extreme biological inflammation (CRP), even when cardinal systemic signs like fever are absent.

The fulminant progression to tetraplegia within hours underscores the narrow therapeutic window in SEA. Surgical decompression is the cornerstone of management for neurological compromise [4]. The clinical progression of SEA traditionally follows four stages: localized spinal pain, radicular pain, motor and sensory deficits, and finally, complete paralysis. However, the speed of this progression is highly variable. While some cases follow a subacute or chronic course over several days or weeks, others-particularly in the cervical spine where the epidural space is more confined-can present a fulminant deterioration. In our patient, the progression to tetraplegia occurred within hours, representing the extreme end of this clinical spectrum. This rapid decline underscores the narrow therapeutic window in acute cases, although clinicians should remain vigilant even in patients with a more indolent presentation, as the transition to irreversible neurological damage can be unpredictable [4].

The decision to avoid immediate stabilization was based on several key criteria. First, the presence of an active, gross pyogenic infection at the C3-C4 level posed a significant risk of bacterial colonization and biofilm formation on any metallic hardware, which could lead to persistent infection and the necessity for subsequent removal [1,4]. Second, spinal stability was deemed sufficiently preserved as the bone destruction was relatively limited, and the surgical approach was tailored to be as bone-sparing as possible.

In the management of cervical SEA, delayed stabilization is often considered a safer alternative. This approach allows for the eradication of the infection through a full course of targeted antibiotic therapy before any reconstructive surgery is undertaken. In our patient, despite the permanent neurological deficits, the four-month follow-up MRI confirmed radiological healing and the maintenance of cervical alignment (Figure 2), thus negating the need for secondary stabilization.

The prognosis for neurological recovery in SEA is influenced by several factors, including the patient’s age, comorbidities (such as diabetes), and the duration and severity of the neurological deficit before surgery [4]. The 'golden window' for surgical intervention is generally considered to be within 24 to 48 hours of the onset of neurological symptoms [4]. However, the severity of the deficit at the time of surgery remains the strongest predictor of outcome. Patients presenting with complete paralysis, especially for more than 24 hours, have a very low probability of recovery [4]. In our case, the deterioration was 'fulminant' rather than 'progressive,' with complete tetraplegia occurring in less than two hours. Despite emergent decompression within this exceptionally narrow timeframe, the lack of recovery underscores that at high cervical levels (C3-C4), the combination of mechanical compression and potential vascular compromise can lead to irreversible cord damage almost instantaneously. This highlights that while early timing is crucial, it cannot always overcome the impact of a devastating initial neurological status.

## Conclusions

In conclusion, cervical SEA remains a life-threatening condition whose clinical presentation can be deceptively mild, particularly in patients treated with TNF-α inhibitors like infliximab. This case highlights that a normal body temperature must never rule out a severe infectious process when other 'red flags' are present. Clinicians should recognize that the combination of acute neck pain and atypical symptoms such as dysphagia, paired with a massive rise in CRP, constitutes a critical warning sign that warrants immediate investigation.

Early diagnosis through urgent MRI is the cornerstone of management, as it allows for immediate surgical decompression. However, as demonstrated in our patient, the therapeutic window in high-cervical SEA can sometimes be exceptionally narrow. Therefore, maintaining a high index of clinical suspicion and prioritizing rapid diagnostic pathways in immunocompromised patients is essential to optimize neurological recovery and prevent permanent disability.
